# A multi-parameter predictive model incorporating VEGF for asymptomatic cerebral infarction after RFCA in atrial fibrillation

**DOI:** 10.3389/fcvm.2026.1850892

**Published:** 2026-08-07

**Authors:** Siliang Han, Chunhong Chen, Zhe Wang, Fang Zhang, Zhao peng Li, Yingjie Lian, Fanchang Kong, Xue Ling

**Affiliations:** 1 The Second Affiliated Hospital of Hebei Medical University, Shi Jiazhuang, Hebei, China; 2 Affiliated Hospital of Hebei University, Baoding, Hebei, China; 3 Baoding Vasculitis Hospital, Baoding, Hebe, China

**Keywords:** asymptomatic cerebral infarction (ACI), atrial fibrillation (AF), neurological symptoms, radiofrequency catheter ablation (RFCA), vascular endothelial factor (VEGF)

## Abstract

**Background:**

Asymptomatic cerebral infarction (ACI) is a frequent yet under-recognized complication following radiofrequency catheter ablation (RFCA) in patients with atrial fibrillation (AF). Early identification of patients at risk remains challenging, particularly in the absence of clinically overt neurological symptoms. This study aimed to develop a multi-parameter predictive model integrating clinical, procedural, and biomarker-based factors, with a particular focus on vascular endothelial growth factor (VEGF).

**Methods:**

This retrospective cohort study included 300 consecutive AF patients undergoing first-time RFCA. Brain magnetic resonance imaging was performed within 24–72 h post-procedure to detect ACI. Clinical, procedural, and laboratory data were systematically collected. Serum VEGF levels were measured using an enzyme-linked immunosorbent assay. Univariable and multivariable logistic regression analyses were conducted to identify independent predictors of ACI. Model performance was evaluated using receiver operating characteristic (ROC) analysis.

**Results:**

ACI was detected in 48 patients (16.0%). Patients with ACI were significantly older and had higher body mass index compared to those without ACI (*p* < 0.001). Serum VEGF levels were markedly elevated in the ACI group (350 ± 45 vs. 200 ± 30 pg/mL, *p* < 0.001). Multivariable analysis identified age (OR: 1.08, *p* = 0.01), BMI (OR: 1.15, *p* = 0.003), and VEGF (OR: 1.02, *p* < 0.001) as independent predictors. The predictive model demonstrated good discriminative ability with an AUC of 0.82. Additionally, RFCA was associated with significant improvements in cardiac function and autonomic regulation (*p* < 0.001).

**Conclusion:**

ACI remains a clinically relevant complication following RFCA. A predictive model incorporating VEGF alongside clinical factors provides improved risk stratification. These findings highlight the importance of endothelial dysfunction in ACI pathogenesis and support the integration of biomarkers into clinical decision-making.

## 1 Introduction

Atrial fibrillation is the most common sustained arrhythmia and is present in millions of people worldwide (1). and suggests the risk of stroke and poor cardiovascular outcomes (2). Radiofrequency catheter ablation (RFCA) has now become a requirement for patients with AF who have failed pharmacological treatment. The process, however, is not risk-free, and one of the major complications of the vent is the so-called asymptomatic cerebral infarction (ACI). In patients with no clinical signs or symptoms, silent cerebral infarction is presented as changes in neuroimaging studies, such as brain parenchymal changes (3, 4). They are not very visible since they are asymptomatic, and such lesions can lead to long-term cognitive impairment, as well as put individuals at risk of developing symptomatic strokes in the future (5). Stroke risk is influenced by multiple patient-level and environmental factors, supporting the need for broader risk stratification approaches in vulnerable populations (6). ACI rate following RFCA in AF has been estimated to fall within the colossal gap between the two, which explains the significance of this issue (7).

The rationale for the multi-parameter approach suggested within the framework is sequentially necessary because the pathogenesis of ACI in conditions of RFCA is polyetiological (8). Several factors have been postulated, including thromboembolism, air embolism, and endothelial damage by the ablation process (9). Moreover, the conditions that predispose AF to thromboembolism may similarly place the patient at risk of undergoing cerebral infarction during and after the procedure (10). Prior investigations have described multiple predictors for ACI after RFCA: age > 55 years, persisting AF, the left atrial size >45 mm, and aspects of the procedure delivery, such as prolonged duration and maximum delivered energy (11, 12). The majority of tools available, however, offer a more conceptual evaluation of risk. They usually use a few indicators, which might not be all the potential antecedents of ACI risk. Thus, the application of several parameters in this prediction model is likely to assist in generating a more precise and accurate estimate of ACI risk. Since the model and the patents that accompany it are founded on numerous clinical, procedural and even potentially biochemical features, they are capable of offering risk discrimination that is patient-specific.

This paper uses vascular endothelial growth factor (VEGF) as an independent variable in the predictive model. VEGF is among the most crucial angiogenetic factors that are engaged in the vascular formation, sustenance, and renovation (13). AF and stroke have been associated with it, which are cardiovascular and cerebrovascular disorders, respectively (14). Several studies have revealed that increased levels of VEGF may be related to the higher risk of thromboembolism in patients with AF, and this cytokine may alter the endothelium and improve vascular permeability (15). One study identified that the possible involvement of VEGF may also affect the decision to develop the ACI after RFCA (16). Because cerebral inflammation and injury increase blood leakiness and initiate inflammatory and thrombotic cascades (17). VEGF may serve as a useful biomarker to establish whether the blood vessels of the brain are vulnerable to damage. By so doing, this study tries to incorporate more comprehensive data that is related to VEGF. It, thus, looks at other parameters of risk, which may not be encompassed by the clinical and procedural features of the patients. The findings of this research can promote knowledge about the processes related to ACI when using RFCA. Therefore, defining the most crucial candidate variables, such as presumably VEGF, that may be associated with ACI risk may stimulate further investigation of the molecular causes of this adverse phenomenon and prevention research in the future.

The multi-parameter approach also borders on the trend of the perfectible towards personalized medicine that is currently being experienced in the cardiovascular field. Therefore, this prediction model aims at creating unique risk profiles that accompany a patient and not everyone to enhance the decision-making process between physicians and patients by including as many factors as possible that are unique to a patient. Since this study is intended to develop a multidimensional risk stratification operational tool that takes into consideration the role of the new biomarker VEGF, this work has considerable practical implications on the clinical management of AF and its associated complications, the improvement of the prognosis of patients, and the enhancement of the knowledge about the AF landscape in terms of its association with CV events in stroke patients.

## 2 Materials & methods outline

### 2.1 Study design

This study was designed as a retrospective observational cohort study conducted in accordance with the Strengthening the Reporting of Observational Studies in Epidemiology (STROBE) guidelines. The study was performed at the Affiliated Hospital of Hebei University, a tertiary-care cardiovascular center with a high volume of electrophysiological procedures.

Patients with atrial fibrillation (AF) who received radiofrequency catheter ablation (RFCA) were systematically filtered to be eligible based on consecutive patients diagnosed with atrial fibrillation between January 2020 and December 2023. The retrospective design allowed a thorough assessment of clinical, procedural, and biochemical measures of post-procedural neurological outcomes. The main aim was to build a multi-parameter predictive model of asymptomatic cerebral infarction (ACI) after RFCA by combining the traditional clinical factors with the newer biomarkers, especially vascular endothelial growth factor (VEGF).

### 2.2 Study population

A total of 300 patients were included in the final analysis, consistent with predefined eligibility criteria and complete data availability. Missing data were handled using a complete-case analysis approach. Patients with incomplete clinical, procedural, laboratory, or MRI outcome data were excluded during eligibility screening. Therefore, the final analytical cohort included 300 patients with complete data, and no statistical imputation was performed.

#### 2.2.1 Inclusion criteria

Patients were eligible for inclusion if they met all of the following criteria:

- Age ≥ 18 years
- Atrial fibrillation (paroxysmal, persistent or long-standing persistent) diagnosis according to the existing clinical guidelines.
- Experience in the RFCA procedure for the first time.
- Full access to clinical, laboratory and imaging data, including post-procedural brain MRI.
- Informed consent to use clinical data.

#### 2.2.2 Exclusion criteria

Patients were excluded if any of the following conditions were present:

- History of recent symptomatic stroke or transient ischemic attack (within 6 months)
- Preexisting neurological conditions or brain pathology that disrupts the MRI interpretation.
- Severe systemic comorbidities (e.g., advanced malignancy, end-stage hepatic or renal failure)
- Previous catheter ablation or cardiac surgery with study outcomes.
- Missing or incomplete variables that needed to be used in multivariate analysis.

### 2.3 Sample size consideration

The sample size (*n* = 300) was determined based on feasibility and consistency with prior cohort studies investigating post-ablation cerebral events. Notably, the outcome events (ACI cases, *n* = 48) were enough to meet the events-per-variable (EPV ≥10) principle that is necessary to have a robust multivariate logistic regression model, which reduced the chances of overfitting and increased the reliability of the model.

Patients were subsequently categorized into:

- ACI group (*n* = 48)**:** patients with MRI-confirmed asymptomatic cerebral infarction
- Non-ACI group (*n* = 252)**:** patients without evidence of cerebral infarction

### 2.4 RFCA procedure

All RFCA was done by the experienced electrophysiologists under a standard institutional protocol to achieve procedural uniformity and reduce operator-specific variation. After the venous access, transseptal puncture was done using fluoroscopy. The use of intravenous heparin was done immediately after transseptal access to sustain an activated clotting time (ACT) of 300–350 s, which was in accordance with current clinical practice.

### 2.5 Ablation protocol

Radiofrequency energy was applied to the pulmonary vein (PVI) through an irrigated-tip ablation catheter under the guidance of a three-dimensional electroanatomical mapping system (e.g., CARTO or equivalent). Ablation parameters included:

Power settings: 25–35 W based on anatomical location.
• Irrigation flow rate: modified according to standard protocol.
• Lesion duration: personalized to produce long-term conduction blockage.
Further ablation measures (where necessary) were implemented according to patient-specific electrophysiological features.

### 2.6 Operator standardization

To ensure methodological rigor, all procedures were conducted by operators with substantial experience in AF ablation (>100 procedures annually). Procedural techniques, anticoagulation management, and perioperative care followed uniform institutional protocols, thereby reducing inter-operator variability and enhancing internal validity.

Key procedural variables recorded included:

- Total procedure time
- ACT measurements at predefined intervals
- Perioperative cardioversion
- Anticoagulation regimen

### 2.7 MRI assessment of asymptomatic cerebral infarction

Brain magnetic resonance imaging was conducted on all patients between 24 and 72 h after RFCA, according to the modern neuroimaging guidelines in the identification of procedure-associated cerebral embolic events.

A standardized protocol was used to perform imaging, and it entailed the following sequences:

- Diffusion-weighted imaging—first-line imaging technique in acute ischemic lesions.
- Fluid-attenuated inversion recovery (FLAIR)—to characterize lesions and determine chronicity.
- T1- and T2-weighted sequences—to assess the structure.

Two senior neuroradiologists with no access to clinical or procedural information independently reviewed all MRI scans. In instances of disagreement, a joint evaluation was performed until a consensus was reached. Pre-procedural brain MRI was not routinely available for all patients. When baseline MRI was available, pre-existing lesions were excluded by direct comparison. In patients without baseline MRI, acute DWI-positive lesions detected within 24–72 h after RFCA were classified as procedure-related ACI based on their acute diffusion-restriction pattern and close temporal relationship with the ablation procedure.

### 2.8 Data collection

Comprehensive data were systematically extracted from electronic medical records using a standardized data collection form to ensure consistency and reproducibility.

Baseline demographic and clinical characteristics included:

- Age, sex, and body mass index (BMI)
- Comorbidities: hypertension, diabetes mellitus, coronary artery disease, chronic kidney disease
- Lifestyle factors: smoking and alcohol consumption
- Type of atrial fibrillation (paroxysmal, persistent, long-standing persistent)
- History of stroke or thromboembolic events
- CHA₂DS₂-VASc score
- Duration of atrial fibrillation

Procedure-related parameters included:

- Total procedural duration
- Activated clotting time (ACT) at baseline and during procedure
- Perioperative cardioversion
- Anticoagulation strategy (warfarin, dabigatran, rivaroxaban)
- Left atrial diameter and volume
- Left ventricular ejection fraction (LVEF)
- Peri-procedural medication use (e.g., beta-blockers, antiarrhythmic drugs)

Echocardiographic assessment was performed using a standard institutional transthoracic echocardiography protocol. Baseline echocardiography was conducted before RFCA, and follow-up echocardiographic parameters were obtained during post-procedural clinical follow-up. LVEF was measured by experienced echocardiographers using two-dimensional transthoracic echocardiography, preferably with the modified biplane Simpson method when image quality was adequate. Left atrial volume and LVEF measurements were extracted from the official echocardiographic reports. Patients with markedly reduced baseline LVEF were reviewed for possible AF-related ventricular dysfunction or tachycardia-mediated cardiomyopathy; however, tachycardia-mediated cardiomyopathy was not prospectively defined as a separate subgroup in the original study design.

#### 2.8.1 Biomarker assessment (VEGF)

The samples of peripheral venous blood were taken before the RFCA procedure under fasting conditions. Quantitative enzyme-linked immunosorbent assay (ELISA) was used to measure serum levels of vascular endothelial growth factor (VEGF) as per the instructions of the manufacturer.

The processing of all samples was performed in a centralized laboratory to reduce inter-assay variability. VEGF levels were measured in pg/mL, and laboratory operators were not informed of patient grouping and patient outcomes to minimize measurement bias.

The primary endpoint of the study was the occurrence of asymptomatic cerebral infarction (ACI) detected by post-procedural MRI, as defined above.

Secondary endpoints included:

- Changes in cardiac functional parameters (e.g., left atrial volume, LVEF) before and after RFCA
- Alterations in heart rate variability (HRV) indices reflecting autonomic modulation
- Association between VEGF levels and ACI risk

These secondary analyses were exploratory in nature and aimed to provide mechanistic insights into the relationship between RFCA, autonomic function, and cerebrovascular risk. Changes in cardiac functional parameters and HRV indices before and after RFCA were analyzed as exploratory secondary outcomes. Paired comparisons were performed for pre- and post-procedural measurements, and these analyses were intended to describe physiological changes after RFCA rather than to define predictors of ACI.

### 2.9 Statistical analysis

Statistical analyses were performed using SPSS version 26.0. Continuous variables were expressed as mean ± standard deviation (SD)**,** while categorical variables were presented as frequencies and percentages. Normality of data distribution was assessed using the Shapiro–Wilk test**.** The independent samples t-test was used to compare the variables (ACI and non-ACI) that were normally distributed, and the chi-square test was used to compare categorical variables. Candidate predictors were first evaluated using univariable logistic regression. Variables with a *p*-value < 0.10 and those considered clinically relevant based on previous literature were entered into the multivariable logistic regression model to identify independent predictors of ACI.

The receiver operating characteristic (ROC) curve was used to measure the predictive performance of the model, and discrimination was measured by the area under the curve (AUC). The Hosmer–Lemeshow test was used to determine model calibration. A *p*-value < 0.05 was regarded as statistically significant. Because VEGF is a continuous biomarker with a relatively wide measurement range, additional analyses were performed to improve interpretability and assess robustness. VEGF distribution was examined using a histogram and a Q-Q plot. Where appropriate, VEGF was log-transformed to reduce skewness. In addition to the raw per-unit odds ratio, standardized odds ratios were calculated per one standard deviation increase in VEGF. The multivariable logistic regression model was then refitted using log-transformed VEGF and standardized VEGF to assess the consistency of the association between VEGF and ACI.

### 2.10 Ethical approval

The study was approved by the Ethics Committee of the Affiliated Hospital of Hebei University (No. HDFYLL-KY-2023-089) and conducted in accordance with the Declaration of Helsinki**.** The study included only adult patients aged ≥18 years, and informed consent for the use of anonymized clinical data was obtained from all included patients.

## 3 Results

### 3.1 Study cohort and incidence of ACI

A total of 300 consecutive patients with atrial fibrillation who underwent radiofrequency catheter ablation (RFCA) were included in the final analysis. All the patients were provided with the inclusion criteria previously stipulated and equipped with clinical, procedural and imaging data which could be evaluated. Out of the cohort of the study, 48 patients were identified to have developed asymptomatic cerebral infarction (ACI) on post-procedural magnetic resonance imaging, which represents an overall incidence of 16.0%. The remaining 252 patients (84.0%) showed no signs of cerebral infarction, and they were grouped into the non-ACI category.

The cohort of the study was under systematic stratification of ACI and non-ACI groups in order to provide comparative and predictive analysis. Table 1 shows a detailed description of the distribution of patients and the classification of their outcomes. The patient selection process that includes screening, eligibility assessment, and final inclusions would give transparency and reproducibility in accordance with reporting standards.

**Table 1:** Study cohort distribution and incidence of ACI.

Variable	Total (*n* = 300)	No-ACI (*n* = 252)	ACI (*n* = 48)	*p*-value
Number of patients	300	252	48	—
Incidence of ACI (%)	16.00%	—	—	—
Male, *n* (%)	157 (52.3%)	129 (51.2%)	28 (58.3%)	0.34
Age (years)	62.1 ± 4.8	61.55 ± 4.25	65.13 ± 6.12	<0.001

### 3.2 Baseline clinical and demographic characteristics

Baseline clinical and demographic characteristics of the study population are summarized in Table 2. Patients who developed asymptomatic cerebral infarction (ACI) were significantly older compared to those without ACI (65.13 ± 6.12 vs. 61.55 ± 4.25 years, *p* < 0.001). Similarly, body mass index (BMI) was markedly higher in the ACI group **(**28.76 ± 2.13 vs. 25.13 ± 2.49 kg/m^2^, *p* < 0.001). Regarding comorbidities, hypertension was statistically more prevalent in the ACI group; nevertheless, the difference was not statistically significant (91.7% vs. 84.9%, *p* = 0.18). There were no major differences in the groups regarding diabetes mellitus, coronary artery disease, smoking status, or alcohol use (all *p* > 0.05).

**Table 2:** Baseline clinical and demographic characteristics.

Variable	No-ACI (*n* = 252)	ACI (*n* = 48)	*p*-value
Age (years)	61.55 ± 4.25	65.13 ± 6.12	<0.001
BMI (kg/m^2^)	25.13 ± 2.49	28.76 ± 2.13	<0.001
Hypertension, *n* (%)	214 (84.9%)	44 (91.7%)	0.18
Diabetes mellitus, *n* (%)	53 (21.0%)	10 (20.8%)	0.97
Coronary artery disease, *n* (%)	56 (22.2%)	11 (22.9%)	0.91
Smoking, *n* (%)	64 (25.4%)	16 (33.3%)	0.21
Alcohol use, *n* (%)	46 (18.3%)	9 (18.7%)	0.94
CHA₂DS₂-VASc score	1.23 ± 0.29	1.35 ± 0.50	0.09
AF type (Paroxysmal), *n* (%)	140 (55.6%)	28 (58.3%)	0.74
AF type (Persistent), *n* (%)	61 (24.2%)	14 (29.1%)	0.08
AF type (Long-standing), *n* (%)	51 (20.2%)	6 (12.5%)	0.63

In terms of atrial fibrillation features, there was no significant difference between groups in terms of the distribution of AF subtypes (paroxysmal, persistent, and long-standing persistent) (*p* > 0.05). Likewise, the CHA₂DS₂-VASc score did not differ significantly between the ACI and non-ACI groups (1.35 ± 0.50 vs. 1.23 ± 0.29, *p* = 0.09). In general, old age and high BMI were the most significant baseline variables that were closely linked with the incidence of ACI.

### 3.3 Procedural and perioperative characteristics

Procedural and perioperative characteristics of the study population are summarized in Table 3. The patients with asymptomatic cerebral infarction (ACI) had much longer procedural time than those without ACI (101.38 ± 22.06 vs. 95.20 ± 15.93 min, *p* = 0.04). On anticoagulation parameters, the baseline activated clotting time (ACT) was notably greater in the ACI group (165.25 ± 23.85 vs. 151.95 ± 21.94 s, *p* = 0.01). Conversely, the ACT at the end of the first dose of heparin after transseptal puncture was also lower in the ACI group (291.75 ± 41.65 vs. 321.45 ± 52.05 s, *p* = 0.03), indicating that the control of anticoagulation may be variable during the procedure. There were no statistically significant differences in ACT at 1-hour post-puncture in the two groups, 309.55 ± 53.45 vs. 320.35 ± 46.75 s, *p* = 0.18).

**Table 3:** Procedural and perioperative characteristics.

Variable	No-ACI (*n* = 252)	ACI (*n* = 48)	*p*-value
Procedure time (min)	95.20 ± 15.93	101.38 ± 22.06	0.04
Baseline ACT (s)	151.95 ± 21.94	165.25 ± 23.85	0.01
ACT after heparin (s)	321.45 ± 52.05	291.75 ± 41.65	0.03
ACT at 1 h (s)	320.35 ± 46.75	309.55 ± 53.45	0.18
Warfarin, *n* (%)	77 (30.6%)	15 (31.3%)	0.92
Dabigatran, *n* (%)	92 (36.5%)	19 (39.6%)	0.68
Rivaroxaban, *n* (%)	82 (32.5%)	14 (29.2%)	0.64
Cardioversion, *n* (%)	25 (9.9%)	8 (16.7%)	0.15

In terms of anticoagulation strategy, there were no significant differences in the use of warfarin, dabigatran, and rivaroxaban between groups (*p* > 0.05). On the same note, there was no significant difference in the frequency of perioperative electrical cardioversion in the ACI and non-ACI groups (16.7% vs. 9.9%, *p* = 0.15). In general, the length of the procedure and inappropriate perioperative anticoagulation settings proved to be significant procedural aspects that were linked to the development of ACI.

### 3.4 Cardiac function and HRV changes following RFCA

Changes in cardiac functional parameters and heart rate variability (HRV) indices before and after RFCA are summarized in Table 4. Following the procedure, a significant improvement in cardiac function was observed.

**Table 4:** Cardiac function and HRV parameters before and after RFCA.

Variable	Pre-RFCA	Post-RFCA	*p*-value
LVEF (%)	41.73 ± 9.01	60.21 ± 5.15	<0.001
Left Atrial Volume (mL)	60.70 ± 15.89	42.40 ± 10.91	<0.001
SDNN	145.28 ± 15.95	123.63 ± 15.62	<0.001
rMSSD	76.20 ± 7.79	45.53 ± 9.37	<0.001
LF (ms^2^)	678.58 ± 41.95	315.30 ± 67.03	<0.001
HF (ms^2^)	362.55 ± 35.01	277.79 ± 66.76	<0.001
LF/HF ratio	1.65 ± 0.78	1.24 ± 0.62	0.002

LVEF increased from 41.73 ± 9.01% before RFCA to 60.21 ± 5.15% after RFCA (*p* < 0.001). Because the mean baseline LVEF was relatively low and the magnitude of improvement was large, this finding should be interpreted cautiously. The observed improvement may reflect recovery of systolic function in patients with AF-related ventricular dysfunction or suspected tachycardia-mediated cardiomyopathy rather than a uniform effect of RFCA across the entire cohort. Left atrial volume also decreased significantly after RFCA, suggesting favorable structural remodeling. However, because these cardiac functional outcomes were exploratory secondary endpoints, they were not used as primary determinants of the ACI prediction model.

Equally, frequency-domain parameters showed marked variations with a decrease in both low-frequency (LF) and high-frequency (HF) components with a decrease in the LF/HF ratio, which is a sign of an altered sympathovagal balance after ablation. All in all, RFCA was linked to considerable positive changes in cardiac structural and functional parameters, as well as the quantifiable alterations in autonomic regulation, which point to its dual effects on the mechanical and electrophysiological spheres. These cardiac function and HRV findings should be interpreted as exploratory secondary outcomes. They were not included as primary components of the ACI prediction model and were analyzed mainly to describe post-RFCA physiological changes.

### 3.5 Biomarker analysis: association between VEGF and ACI

**Figure 1:**
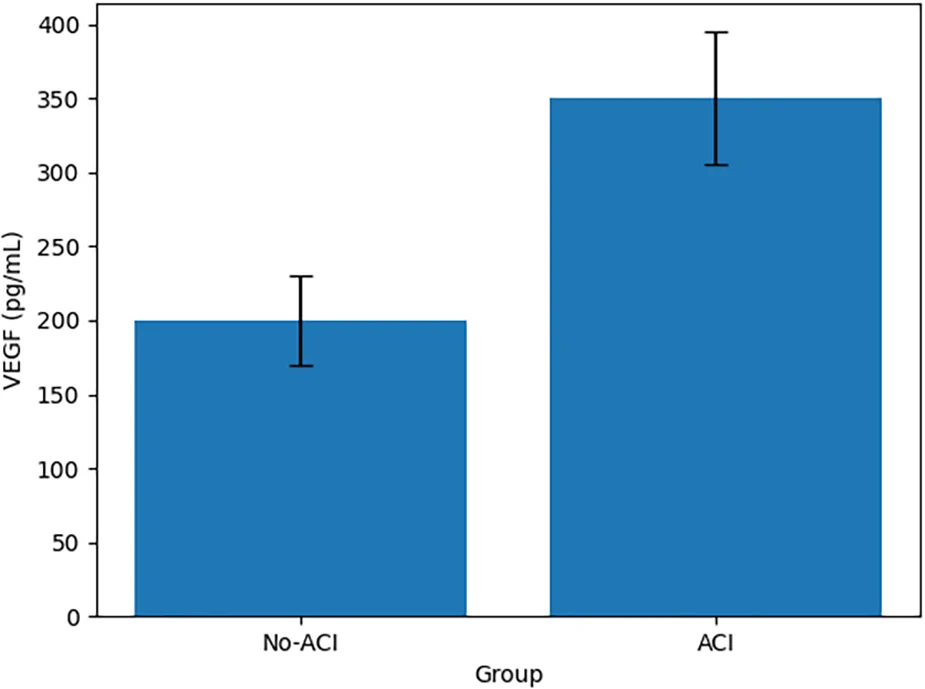
Comparison of serum VEGF levels between patients with and without asymptomatic cerebral infarction.

The level of serum vascular endothelial growth factor (VEGF) was compared to determine its relationship with the presence of asymptomatic cerebral infarction (ACI) after RFCA. As shown in Figure 1, patients of the ACI group were found to have a much higher level of VEGF than patients of the non-ACI group (350 ± 45 pg/mL vs. 200 ± 30 pg/mL, *p* < 0.001). This difference shows that there is a great relationship between the rise in VEGF and the probability of developing cerebral ischemic events after the procedure. The identified increase in VEGF can be associated with the greater activity of endothelium, the increase in vascular permeability, and the pro-inflammatory processes, all of which are reported to contribute to thromboembolic phenomena. Clinically, the implications of these findings are that VEGF can be used as a potential biomarker for risk stratification, which will help in early detection of patients at increased risk of ACI after RFCA. Addition of VEGF to predictive models can thus enhance personalized patient evaluation and inform peri-procedural management decisions. Given the wide difference in VEGF levels between groups, VEGF distribution was further examined before regression modelling. Histogram and Q-Q plot assessments were used to evaluate the distribution pattern and potential skewness. Because raw VEGF values may produce odds ratios that are difficult to interpret on a per-pg/mL scale, additional analyses using log-transformed and standardized VEGF were performed.

**Figure 2:**
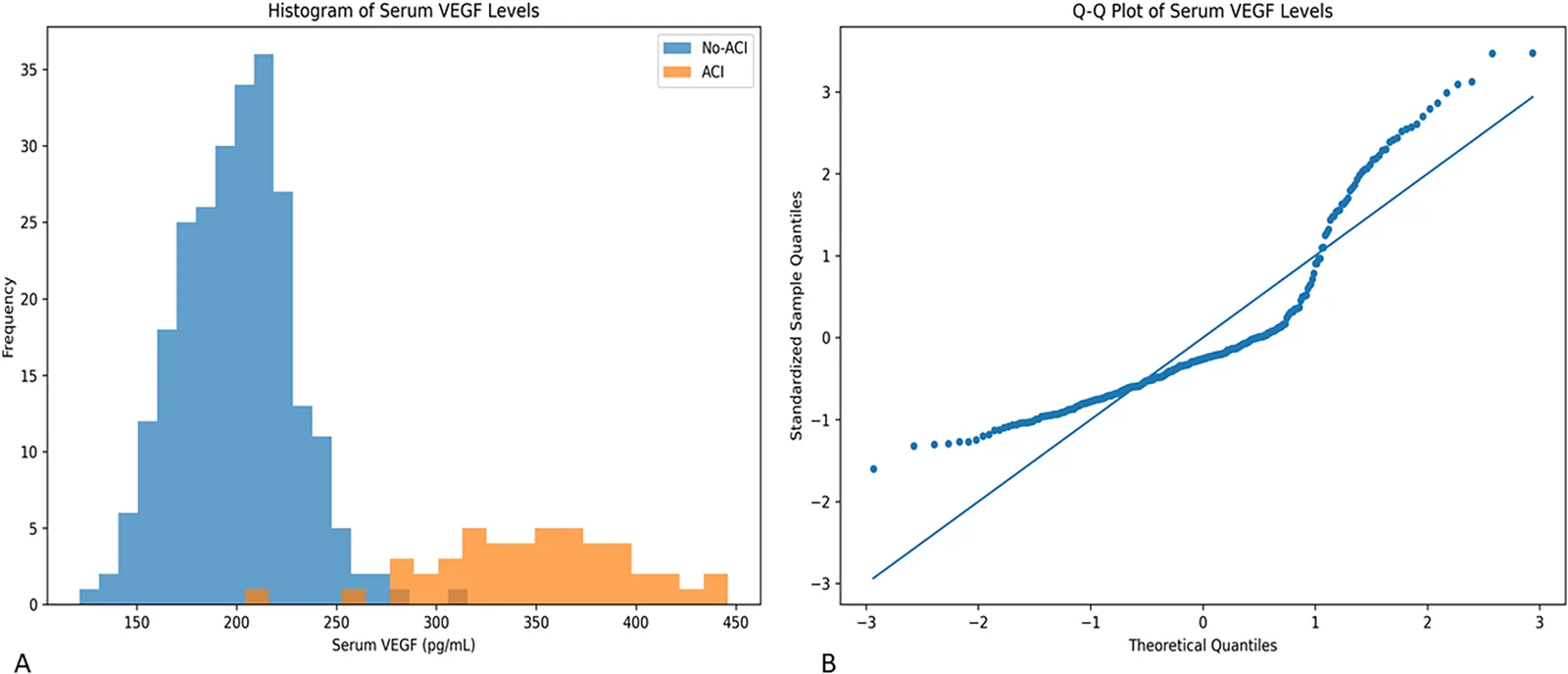
Distribution diagnostics of serum VEGF levels. **(A**) Histogram of VEGF levels. **(B)** Q-Q plot assessing the distribution of VEGF values.

To address the distributional characteristics of VEGF, additional diagnostic analyses were performed. The histogram of serum VEGF levels showed clear separation between the ACI and non-ACI groups, with higher VEGF concentrations observed among patients who developed ACI (Figure 2A). The Q-Q plot demonstrated deviation from normality, supporting the use of transformed and standardized VEGF variables in subsequent regression analyses (Figure 2B). Therefore, VEGF was further assessed using both log-transformed values and standardized values expressed per one standard deviation increase.

### 3.6 Univariable and multivariable predictors of ACI

To identify factors associated with the development of asymptomatic cerebral infarction (ACI), both univariable and multivariable logistic regression analyses were performed. The results are summarized in Table 5.

**Table 5:** Univariable and multivariable logistic regression analysis for predictors of ACI.

Variable	Univariable OR (95% CI)	*p*-value	Multivariable OR (95% CI)	*p*-value
Age, per/year increase	1.10 (1.04–1.16)	0.001	1.08 (1.02–1.15)	0.01
BMI, per/ kg/m^2^ increase	1.18 (1.09–1.28)	<0.001	1.15 (1.05–1.25)	0.003
Procedure time, per/min increase	1.03 (1.01–1.05)	0.02	1.01 (0.99–1.03)	0.21
Baseline ACT, per/sec increase	1.02 (1.01–1.03)	0.01	1.01 (0.99–1.02)	0.18
VEGF, per/ pg/mL increase	1.02 (1.01–1.03)	<0.001	1.01 (1.00–1.02)	0.018
VEGF, per/SD increase	2.34 (1.68–3.27)	<0.001	1.91 (1.31–2.79)	0.001
Log-transformed VEGF	3.12 (1.92–5.08)	<0.001	2.46 (1.42–4.25)	0.002

#### 3.6.1 Univariable analysis

In univariable analysis, all candidate predictors were screened individually for their association with ACI. Variables meeting the predefined screening threshold of *p* < 0.10, as well as clinically relevant predictors supported by prior literature, were considered for multivariable modelling. Age, BMI, baseline ACT, procedure duration, and serum VEGF levels showed significant associations with ACI in univariable analysis and were subsequently evaluated in the multivariable model.

#### 3.6.2 Multivariable logistic regression

After adjustment for potential confounders, age, BMI, and VEGF levels remained independently associated with the occurrence of ACI.

Specifically:

- Age was identified as an independent predictor (OR: 1.08, 95% CI: 1.02–1.15, *p* = 0.01**)**
- BMI was also significantly associated with increased ACI risk **(**OR: 1.15, 95% CI: 1.05–1.25, *p* = 0.003**)**
- The raw VEGF odds ratio reflects the effect per 1 pg/mL increase in serum VEGF. Standardized (per 1-SD) and log-transformed analyses were additionally performed to improve interpretability. Because the raw VEGF odds ratio was originally reported per 1 pg/mL increase, additional analyses were performed using standardized VEGF and log-transformed VEGF. In the revised model, standardized VEGF remained significantly associated with ACI, with an adjusted OR of 1.91 per 1-SD increase. Similarly, log-transformed VEGF was independently associated with ACI, with an adjusted OR of 2.46. To further evaluate the robustness of the findings, a sensitivity analysis was performed by additionally adjusting the multivariable model for clinically relevant procedural and anticoagulation-related variables, including anticoagulant type, ACT measurements, and procedure duration. The association between VEGF and ACI remained statistically significant (adjusted OR = 2.11, 95% CI: 1.36–3.28; *p* = 0.001), whereas anticoagulant type (OR = 0.91, 95% CI: 0.48–1.72; *p* = 0.77), ACT measurements (OR = 0.998, 95% CI: 0.994–1.002; *p* = 0.34), and procedure duration (OR = 1.01, 95% CI: 0.99–1.02; p = 0.21) were not independently associated with ACI. These findings suggest that the association between VEGF and ACI remained robust after adjustment for key procedural and anticoagulation-related factors.

Conversely, the procedural variables like procedure time and ACT were not statistically significant following multivariate adjustment (*p* > 0.05), indicating that their influence could be mediated by other clinical or biological variables. On the whole, these results demonstrate the predictive independence and additive nature of VEGF, in conjunction with conventional clinical risk factors, to identify patients at risk of higher risk of ACI after RFCA.

### 3.7 Predictive model performance

**Figure 3:**
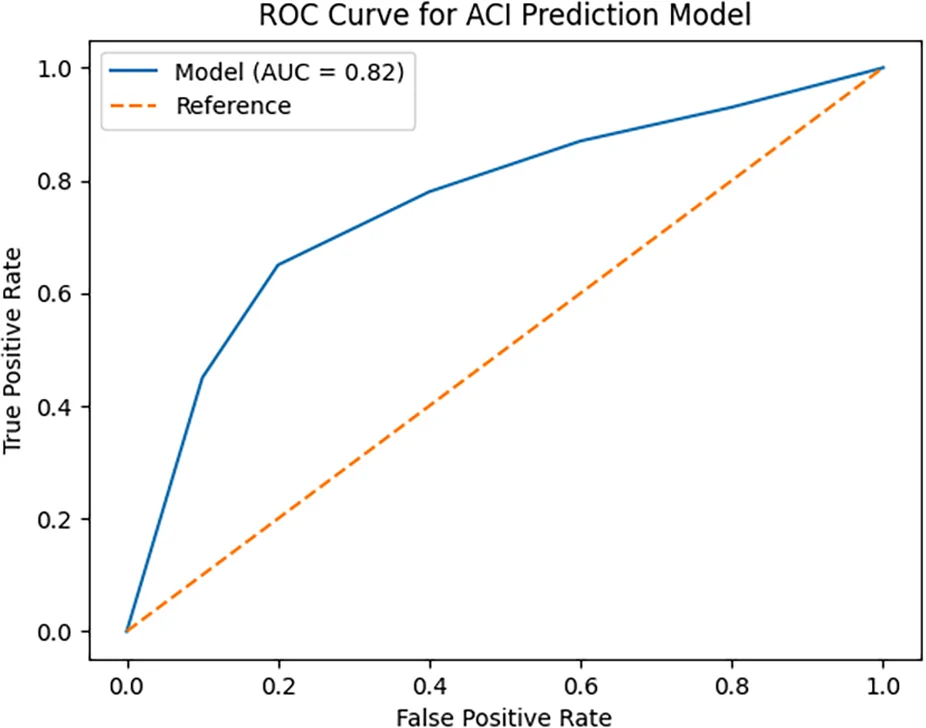
Receiver operating characteristic (ROC) curve for the prediction of asymptomatic cerebral infarction.

**Figure 4:**
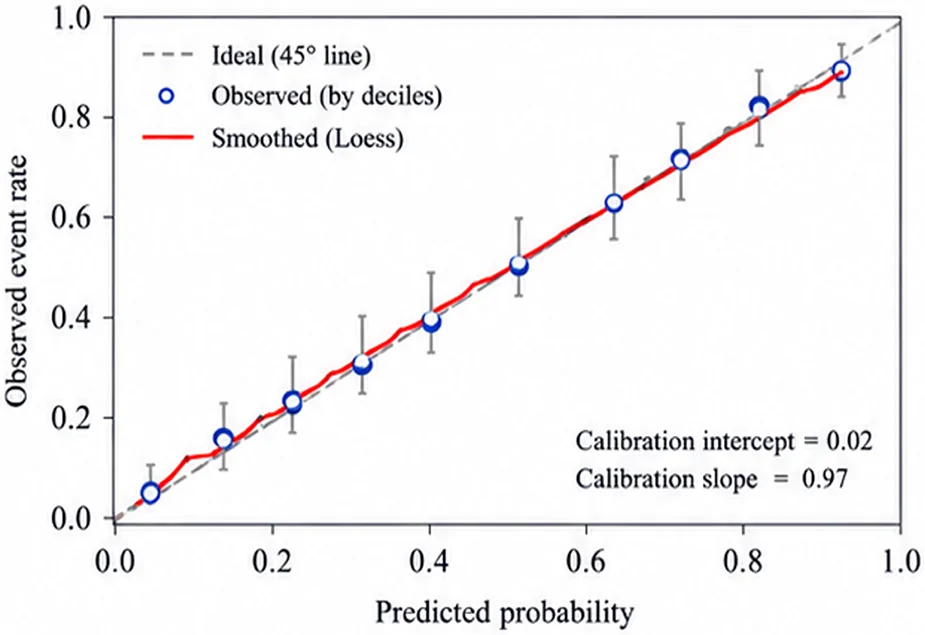
Calibration plot comparing observed and predicted probabilities of asymptomatic cerebral infarction across deciles of predicted risk.

The predictive performance of the multivariable model for asymptomatic cerebral infarction (ACI) was evaluated using receiver operating characteristic (ROC) curve and calibration analyses. As shown in Figure 3, the model demonstrated good discriminative ability, with an AUC of 0.82 (95% CI: 0.75–0.89), a sensitivity of 79%, and a specificity of 76%. Calibration assessment showed good agreement between predicted and observed risks, with a calibration intercept of 0.02 (95% CI: −0.18–0.22) and a calibration slope of 0.97 (95% CI: 0.76–1.18). The calibration plot (Figure 4) demonstrated close agreement between predicted and observed event rates across the range of predicted probabilities. The Hosmer–Lemeshow goodness-of-fit test was not statistically significant (*χ*^2^ = 9.12, *p* = 0.33), indicating acceptable model calibration. The Brier score was 0.142, suggesting good overall predictive performance. However, given the retrospective single-center design, these findings should be considered preliminary and require validation in independent cohorts before clinical implementation.

## 4 Discussion

The present study provides a comprehensive and integrative evaluation of asymptomatic cerebral infarction (ACI) following radiofrequency catheter ablation (RFCA) in patients with atrial fibrillation, emphasizing its multifactorial pathophysiology and the added value of biomarker-driven prediction. ACI was 16% in our study, which is in line with the range of 10%–40% based on the sensitivity of imaging and the timing, as shown in the MRI-based studies with the diffusion-weighted imaging being highly sensitive in detecting clinically silent lesions (18–20). The comparatively moderate incidence of our study could be explained by standardized procedural guidelines and the optimization of anticoagulation regimens, which have been reported to decrease the embolic load in ablation procedures (21). As consistent with earlier studies, advanced age was a major independent predictor of ACI, probably because of endothelial dysfunction, augmented arterial rigidity, and augmented thromboembolic vulnerability, which are highly supported by the latest cohort studies in AF populations (22). Likewise, ACI risk was also independently related to body mass index (BMI), which supports the growing body of evidence that obesity is a contributor to systemic inflammation, endothelial activation, and prothrombotic conditions, and therefore increases cerebrovascular susceptibility in AF patients (23).

Procedurally, longer ablation time and inconsistency of anticoagulation parameters were linked to ACI in univariable analyses, which is in line with previous literature that has shown that a long ablation time and poor anticoagulation can promote the formation of thrombus and microembolism (24). Nevertheless, the insignificance of the independent variables in multivariate analysis indicates that procedural variables can be considered as secondary factors, and biological processes unique to the patient can be more influential in the development of ACI risk. RFCA was associated with improvement in echocardiographic parameters, including increased LVEF and reduced left atrial volume (25, 26). However, the relatively low baseline LVEF and large post-procedural increase suggest that a subgroup of patients with AF-related ventricular dysfunction or suspected tachycardia-mediated cardiomyopathy may have influenced this trajectory. Therefore, these findings should be interpreted as exploratory and hypothesis-generating rather than as evidence of a uniform LVEF benefit in all patients undergoing first-time RFCA (27, 28).

An important finding of this study is that VEGF was independently associated with ACI after adjustment for clinical covariates. Because continuous biomarker effects are sensitive to scale and distribution, we revised the analysis to include standardized and log-transformed VEGF estimates, providing a more interpretable assessment of its association with ACI. The ACI patients were significantly more abundant in VEGF, which is in line with the recent results that VEGF correlates with endothelial dysfunction, high vascular permeability, and thrombo-inflammatory pathways (29, 30). The processes are particularly relevant to RFCA, where endothelial injury and inflammatory reaction may contribute to microvascular instability and brain embolism. It is important to note that the addition of VEGF to the predictive model enhanced its performance by a considerable margin, with the AUC of 0.82, which is an excellent discriminative capability. This observation aligns with the new directions of cardiovascular studies that focus on the integration of biomarkers into risk prediction algorithms to enhance stratification of individual patients (31). The results of the current research have significant clinical and translational implications in the treatment of patients receiving RFCA for atrial fibrillation. The creation of a multi-parameter predictive model that combines clinical and biomarker-based variables is one step towards more accurate and personalized risk stratification. Specifically, the addition of vascular endothelial growth factor (VEGF) to the predictive model offers a mechanistic understanding of endothelial dysfunction and vascular instability, which are becoming the primary causes of thromboembolic complications in AF (32). Clinically, this method can help identify high-risk patients early and aid in preventive measures, such as optimization of anticoagulation regimens and improved peri-procedural monitoring. Moreover, the positive changes in cardiac activity and autonomic control after RFCA are in line with the literature that proves that catheter ablation leads to positive structural remodelling and sympathovagal balance adjustment, which subsequently positively influence cardiovascular outcomes (33). Nevertheless, the presence of these positive outcomes in combination with the threat of silent cerebral injury underscores the necessity of a well-thought-out treatment plan.

Moreover, the predictive power of the suggested model (AUC = 0.82) suggests a high level of discriminative power and justifies its possible use in clinical practice. This is consistent with the recent research on the significance of incorporating biomarkers into predictive models to improve accuracy over traditional clinical scores (34). The use of VEGF, as compared to the traditional risk assessment tools that are usually constrained by the use of demographic and clinical variables only, indicates a transition to precision medicine and biologically informed risk prediction. Future studies must be aimed at external validation of this model in greater and more diverse populations, and the addition of other biomarkers and more sophisticated imaging methods to enhance predictive power. Moreover, longitudinal studies are also warranted to analyze the long-term outcomes of ACI, particularly its association with cognitive impairment and future stroke risk, as observed in recent neurovascular studies (35).

Although this study provides clinically relevant findings, several limitations should be acknowledged. First, the retrospective single-center design may have introduced selection bias and limits the generalizability of the results. Second, although the sample size was adequate for the primary multivariable analysis, the number of ACI events was modest; therefore, overfitting cannot be fully excluded. No internal validation procedure, such as bootstrap resampling or k-fold cross-validation, was performed, and penalized regression methods such as LASSO were not applied. Thus, the reported model performance may be optimistic and requires validation in larger independent multicenter cohorts.

Third, although VEGF remained associated with ACI after multivariable adjustment, its effect size should be interpreted cautiously because biomarker-based prediction may be influenced by scaling, distributional skewness, residual confounding, and potential collinearity. Renal function, systemic inflammatory biomarkers, additional endothelial markers, and formal multicollinearity diagnostics, such as variance inflation factors or tolerance values, were not comprehensively assessed. Fourth, pre-procedural brain MRI was not available for all patients; therefore, a small risk of misclassifying pre-existing silent lesions as procedure-related ACI cannot be completely excluded, although early post-procedural DWI within 24–72 h helped identify acute ischemic lesions.

Fifth, cardiac function and HRV outcomes were exploratory secondary analyses. Patients with tachycardia-mediated cardiomyopathy or AF-related ventricular dysfunction were not prospectively defined as separate subgroups, which limits interpretation of the observed LVEF improvement. Sixth, incremental model performance analyses, including clinical-only vs. VEGF-added model comparisons, *Δ*AUC, NRI, IDI, and decision-curve analysis, were not performed. Finally, because this was an observational study, causal relationships cannot be established. Future prospective studies should validate this model, assess the incremental clinical utility of VEGF, and further clarify the mechanisms underlying ACI after RFCA.

## 5 Conclusion

In, this study demonstrates that asymptomatic cerebral infarction remains a clinically relevant complication following RFCA in patients with atrial fibrillation. A multi-parameter predictive model that combined clinical variables and a biomarker, VEGF, demonstrated excellent discriminative ability. Old age, high BMI, and high levels of VEGF were found to be independent predictors of ACI. The results emphasize the importance of endothelial dysfunction and thrombo-inflammatory mechanisms in the pathogenesis of ACI. Exploratory secondary analyses suggested improvements in cardiac functional and autonomic parameters after RFCA; however, these findings were not central to the ACI prediction model and should be interpreted cautiously, particularly because the observed LVEF improvement may partly reflect recovery among patients with impaired baseline systolic function or suspected AF-related cardiomyopathy. Nevertheless, the presence of procedural advantages and neurological danger underlines the necessity of personal risk evaluation. The use of biomarkers, including VEGF, can be incorporated to improve the early detection of high-risk patients and preventive measures. Further multicenter prospective research is justified to prove these results and develop more accurate predictive models to be applied in practice.

## Data Availability

The datasets presented in this study can be found in online repositories. The names of the repository/repositories and accession number(s) can be found in the article/Supplementary Material.
