# Supplementary material for: A multi-parameter predictive model incorporating VEGF for asymptomatic cerebral infarction after RFCA in atrial fibrillation
Source: Front Cardiovasc Med. 2026 Aug 7;13:1850892. doi: 10.3389/fcvm.2026.1850892 (PMC13494712; doi:10.3389/fcvm.2026.1850892)
Supplement: Supplementary file 1 [file Table1.docx]

**Supplementary Table**

Supplementary Table S1. Full univariable logistic regression analysis of candidate predictors for asymptomatic cerebral infarction

| **Candidate predictor** | **Univariable OR** | **95% CI** | **p-value** |
| --- | --- | --- | --- |
| Age, per 1-year increase | 1.1 | 1.04–1.16 | 0.001 |
| Sex, male vs. female | 1.34 | 0.72–2.49 | 0.43 |
| BMI, per 1 kg/m² increase | 1.18 | 1.09–1.28 | <0.001 |
| Hypertension, yes vs. no | 1.95 | 0.66–5.75 | 0.26 |
| Diabetes mellitus, yes vs. no | 0.99 | 0.46–2.11 | 1 |
| Coronary artery disease, yes vs. no | 1.04 | 0.50–2.17 | 1 |
| Smoking, yes vs. no | 1.47 | 0.76–2.85 | 0.29 |
| Alcohol use, yes vs. no | 1.03 | 0.47–2.28 | 1 |
| CHA₂DS₂-VASc score, per 1-point increase | 2.92 | 1.15–7.38 | 0.024 |
| AF type, persistent vs. paroxysmal | 1.8 | 0.96–3.40 | 0.09 |
| AF type, long-standing persistent vs. paroxysmal | 1.09 | 0.52–2.30 | 0.85 |
| Procedure time, per 1-min increase | 1.03 | 1.01–1.05 | 0.02 |
| Baseline ACT, per 1-sec increase | 1.02 | 1.01–1.03 | 0.01 |
| ACT after heparin, per 1-sec increase | 0.99 | 0.98–0.99 | 0.03 |
| ACT at 1 hour, per 1-sec increase | 1 | 0.99–1.00 | 0.15 |
| Warfarin use, yes vs. no | 1.03 | 0.53–2.01 | 1 |
| Dabigatran use, yes vs. no | 1.14 | 0.61–2.15 | 0.75 |
| Rivaroxaban use, yes vs. no | 0.85 | 0.43–1.68 | 0.74 |
| Cardioversion, yes vs. no | 1.82 | 0.77–4.31 | 0.21 |
| VEGF, per 1 pg/mL increase | 1.02 | 1.01–1.03 | <0.001 |
| VEGF, per 1-SD increase | 2.34 | 1.68–3.27 | <0.001 |
| Log-transformed VEGF | 3.12 | 1.92–5.08 | <0.001 |

Supplementary Table S2. Sensitivity analysis of predictors of asymptomatic cerebral infarction after adjustment for procedural and anticoagulation-related variables

| **Predictor** | **Adjusted OR** | **95% CI** | **P-value** |
| --- | --- | --- | --- |
| VEGF (per 1-SD increase) | **2.11** | **1.36–3.28** | **0.001** |
| Age | 1.03 | 1.00–1.06 | 0.041 |
| BMI | 1.08 | 1.01–1.16 | 0.028 |
| Hypertension | 1.47 | 0.82–2.64 | 0.19 |
| Anticoagulant type (DOAC vs Warfarin) | 0.91 | 0.48–1.72 | 0.77 |
| ACT (1 hour) | 0.998 | 0.994–1.002 | 0.34 |
| Procedure duration | 1.01 | 0.99–1.02 | 0.21 |
